# Identification of *GREM-1* and *GAS6* as Specific Biomarkers for Cancer-Associated Fibroblasts Derived from Patients with Non-Small-Cell Lung Cancer

**DOI:** 10.3390/cancers17172858

**Published:** 2025-08-30

**Authors:** Bo-Guen Kim, Kyunghee Park, Mina Hwang, Hyewon Lee, Kyung-Mi Park, Junsu Choe, Sun Hye Shin, Byeong-Ho Jeong, Kyungjong Lee, Junghee Lee, Yeong Jeong Jeon, Jong Ho Cho, Hong Kwan Kim, Woong-Yang Park, Sang-Won Um

**Affiliations:** 1 Division of Pulmonary and Critical Care Medicine, Department of Medicine, Samsung Medical Center, Sungkyunkwan University School of Medicine, Seoul 06351, Republic of Korea; boguen86.kim@samsung.com (B.-G.K.); mn.hwang@sbri.co.kr (M.H.); gyeong.mi@sbri.co.kr (K.-M.P.); junsu.choe@samsung.com (J.C.); fresh.shin@samsung.com (S.H.S.); bh82.jeong@samsung.com (B.-H.J.); kj2011.lee@samsung.com (K.L.); 2 Division of Pulmonary Medicine, Department of Internal Medicine, Kangbuk Samsung Hospital, Sungkyunkwan University School of Medicine, Seoul 03181, Republic of Korea; 3 Samsung Genome Institute, Samsung Medical Center, Seoul 06351, Republic of Korea; kyunghee.park@samsung.com (K.P.); woongyang.park@samsung.com (W.-Y.P.); 4 Department of Obstetrics and Gynecology, Seoul St. Mary’s Hospital, College of Medicine, The Catholic University of Korea, 222, Banpo-daero, Seocho-gu, Seoul 06591, Republic of Korea; hyewon153.lee@catholic.ac.kr; 5 Department of Thoracic and Cardiovascular Surgery, Samsung Medical Center, Sungkyunkwan University School of Medicine, Seoul 06351, Republic of Korea; jhts.lee@samsung.com (J.L.); ts.yj.jeon@samsung.com (Y.J.J.); jongho9595.cho@samsung.com (J.H.C.); hkts@skku.edu (H.K.K.); 6 Department of Health Sciences and Technology, SAIHST, Sungkyunkwan University, Seoul 06351, Republic of Korea

**Keywords:** non-small cell carcinoma, cancer-associated fibroblast, lymph node, lung

## Abstract

**Background/Objectives:** Cancer-associated fibroblasts (CAFs) play a pivotal role in the tumor microenvironment. We conducted an analysis using RNA sequencing to identify specific markers for CAFs compared to normal fibroblasts (NFs) in non-small-cell carcinoma (NSCLC). **Methods:** CAFs and NFs were isolated and cultured from tumor tissues (primary tumor or metastatic lymph nodes) and matched non-tumor tissues, respectively. Bulk RNA sequencing was conducted on isolated CAFs and normal fibroblast NFs. Differential expressions, gene set enrichment, and CAF subpopulation prediction analyses were performed. **Results:** During the study period, 27 CAFs and 12 NFs were isolated and cultured from tumor and non-tumor tissues in patients with treatment-naïve NSCLC. Among them, 22 CAFs and 11 NFs were included in the RNA sequencing analysis. The 22 CAF samples consisted of 12 adenocarcinomas and 10 squamous cell carcinomas (SqCC), with 16 samples from the lungs and 6 samples from the lymph nodes. Notably, *COL11A1*, *GREM1*, *CD36*, and *GAS6* showed a higher expression in CAFs than in NFs, whereas *TNC* and *CXCL2* were more abundantly expressed in NFs. *CD36* levels were elevated in CAFs from lymph nodes (LN-CAFs) compared with those from lung specimens (Lung-CAFs) and NFs. *COL11A1* levels in Lung-CAFs surpassed those in LN-CAFs and NFs. Both *GREM1* and *GAS6* showed a strong expression in Lung-CAFs and LN-CAFs relative to NFs. CAFs exhibited features of the myofibroblast CAF subpopulation, whereas NFs displayed traits of the antigen-presenting CAF subtype. In the co-culture model of CAFs and THP-1 cells, the knockdown of *GREM1* or *GAS6* in CAFs significantly decreased the M2 marker expression in macrophages. **Conclusions:** In NSCLC, *GREM1* and *GAS6* can be valuable diagnostic targets for CAFs from primary tumors and metastatic sites; they warrant further study.

## Simple Summary

Cancer-associated fibroblasts (CAFs) play a crucial role in the tumor stroma. Our RNA sequencing analysis with 22 CAF and 11 normal fibroblast (NF) samples of non-small-cell carcinoma (NSCLC) revealed specific CAF markers. *COL11A1*, *GREM1*, *CD36*, and *GAS6* are highly expressed in CAFs. Both *GREM1* and *GAS6* showed a strong expression in CAFs from lymph nodes and CAFs from lung specimens relative to NFs. *TNC* and *CXCL2* are prominent in NFs. Differential expression patterns were observed in lymph node and lung specimens. In the co-culture model of CAFs and THP-1 cells, the knockdown of *GREM1* or *GAS6* in CAFs significantly decreased the M2 marker expression in macrophages. In NSCLC, GREM1 and GAS6 represent potential diagnostic targets for CAFs derived from both primary tumors and metastatic sites.

## 1. Introduction

CAFs are fibroblasts that reside within the tumor microenvironment and have been activated or reprogrammed by tumor-derived signals, while normal fibroblasts (NFs) are fibroblasts found in non-tumor tissues that maintain tissue homeostasis and normal wound healing [1,2]. Unlike CAFs, NFs do not actively promote tumor growth or immune evasion [1,2]. CAFs, also known as tumor-surrounding fibroblasts, constitute a key segment of the tumor stroma [1,3]. Their pivotal role in promoting tumor growth and progression is mediated through various mechanisms, including the secretion of growth factors, chemokines, and extracellular vesicles, as well as participation in extracellular matrix (ECM) remodeling [4]. This coordination effectively regulates key aspects of tumor behavior, such as proliferation, metastatic potential, chemotherapy resistance, immune evasion, and reactivation from dormancy [1,3,5,6,7].

Since the identification of CAFs, advances in immunohistochemical methods and single-cell RNA sequencing have redefined classical CAF markers, including the platelet-derived growth factor receptor-α/β, α-smooth muscle actin (αSMA), fibroblast activation protein (FAP), and fibroblast-specific protein-1 [5,8,9,10,11,12,13]. However, these proteins are also found in various immune cells and certain cancer cells [9,11,14,15]. A technical challenge in isolating CAFs is the lack of specific markers because most known markers are shared with other cell types. For example, podoplanin is expressed in lymphatic endothelial cells [16], FAP is expressed in macrophages [17], and αSMA is predominantly expressed in smooth muscle cells [18]. These aspects highlight the need for a collection of subtype-specific markers, distinct from those of NFs, to accurately identify various CAF populations. Furthermore, whereas CAF markers have been explored in many cancers, NSCLC-specific CAF markers remain limited. Moreover, the relationships and functions between CAFs at metastatic and primary sites remain unclear.

The aims of this study were to analyze and identify distinct markers indicative of CAFs compared with NFs and to investigate the subpopulations of CAFs and NFs in NSCLC.

## 2. Materials and Methods

### 2.1. Study Subjects and Study Samples

Study subjects included the patients with NSCLC. Lung-origin CAFs (Lung-CAFs) and NFs were derived from resected primary lung tumors and non-tumorous lungs, respectively. Lymph node-origin CAFs (LN-CAFs) were derived from metastatic lymph nodes obtained by endobronchial ultrasound-guided transbronchial needle aspiration (EBUS-TBNA). The study protocol was approved by the Institutional Review Board of Samsung Medical Center (IRB No. 2014-03-140). All participants provided informed consent before any study procedures. Our study complied with the Declaration of Helsinki.

### 2.2. Isolation and Culture of CAFs and NFs

Isolation and culture of CAFs and NFs were described in our previous report [19]. In this study, fibroblasts isolated and cultured from tumor tissues were classified as CAFs, while those derived from non-tumor tissues were classified as NFs. CAFs were isolated from tumor tissues (primary tumor or metastatic lymph nodes), and NFs were isolated from the most distant non-tumor tissue from the resected lungs of NSCLC patients. Non-tumor and tumor specimens were washed three times with phosphate-buffered saline (PBS; HyClone, Logan, UT, USA). Tissue specimens of primary lung tumors, metastatic lymph nodes, and non-tumorous lung tissues were minced into 1–2-mm^3^ pieces and digested using 1 mL of 0.25% collagenase type I (STEMCELL Technologies, Vancouver, BC, Canada) at 37 °C in an incubator for 30 min to 1 h until the samples comprised > 70% single cells. After enzyme digestion, samples were centrifuged at 1300 rpm for 3 min. Pellets were resuspended in DMEM/F12 (Gibco, Invitrogen, Carlsbad, CA, USA), 10% fetal bovine serum (FBS; Gibco), and 1% antibiotic-antimycotic solution (Gibco). Next, pellets were placed in 100 mm dishes. Cells were expanded for 7–14 days until they reached 90% confluence. Cells that migrated from seeded tissue fragments were collected after treatment with Trypsin-EDTA (Gibco). The collected cells were passed through a 100 µm strainer (Falcon, Franklin Lakes, NJ, USA) and spread in 100 mm dishes. The cells were then expanded to passage 3 and cryopreserved in complete DMEM with 10% dimethyl sulfoxide (Sigma-Aldrich, St. Louis, MO, USA) until analysis. CAFS and NFs were validated by the absence of E-cadherin expression in RT-PCR and an EpCAM expression level below 1% in flow cytometry.

### 2.3. Real-Time Polymerase Chain Reaction (RT-PCR)

Total RNA was isolated from fibroblasts using the RNeasy Mini Kit (Qiagen, Hilden, Germany) according to the manufacturer’s instructions. RNA concentration and purity were assessed using a Nanodrop 2000 spectrophotometer (ThermoFisher Scientific, Waltham, MA, USA). Complementary DNA (cDNA) was synthesized from total RNA using the SuperScript III First-Strand Synthesis System (ThermoFisher Scientific). Primer sequences used in the study are listed in Table S1. The H1975 NSCLC cell line (ATCC^®^ CRL-5908™) was used as a positive control for epithelial cell contamination, given its known expression of epithelial markers such as E-cadherin. The IMR90 normal fibroblast cell line (ATCC^®^ CCL-186™) was used as a positive control for fibroblasts.

### 2.4. Flow Cytometry Analysis

For surface marker analysis, cells were washed and blocked with FACS buffer (1% bovine serum albumin in PBS), followed by incubation with fluorophore-conjugated antibodies at 4 °C for 20 min. Antibody information is detailed in Table S2. Prior to acquisition, single-cell suspensions were prepared by filtering through a 35 µm cell strainer cap (BD Biosciences). Samples were analyzed using the FACSVerse cytometer and FACSVerse/FACSuite v1.5 software (BD Biosciences, San Jose, CA, USA).

### 2.5. RNA Sequencing Analysis

The total RNA from cultured cell pellets of CAFs or NFs was extracted with an RNeasy Mini Kit (Qiagen). Nucleic acid extraction was performed according to the manufacturer’s instructions. The quality and quantity of extracted nucleic acids were evaluated using a Nanodrop 8000 UV–Vis spectrometer (NanoDrop Technologies Inc., Wilmington, DE, USA), Qubit^®^ 3.0 Fluorometer (Life technologies, Inc., Carlsbad, CA, USA), and 4200 TapeStation (Agilent Technologies, Inc., Santa Clara, CA, USA). Sequencing libraries were prepared with a TruSeq RNA Sample Preparation Kit v2, set A and B (Illumina, Inc., San Diego, CA, USA, Cat# RS-122-2001 and RS-122-2002) following manufacturer’s protocols. Paired-end sequencing (2 × 100 bp) of the RNA libraries was performed on a HiSeq 2500 Sequencing Platform (Illumina, Inc.). The average sequencing depth was 81 million paired-end reads per sample (range: 66–126 million). After trimming poor quality bases from the FASTQ files, reads were aligned to the human reference genome (hg19) with STAR v2.5.2b (https://github.com/alexdobin/STAR [accessed on 30 August 2025]) [20], and estimated gene expression was calculated in terms of estimated expected counts using RSEM v1.3 (https://github.com/deweylab/RSEM [accessed on 30 August 2025]) [21].

### 2.6. Differential Expression and Gene Set Enrichment Analysis

Differential expressions (DEs) were performed using generalized linear modeling through the glmFit function in the R package egdeR v3.36.1 (https://bioconductor.org/packages/release/bioc/html/edgeR.html [accessed on 30 August 2025]) [22] for comparison between CAFs and NFs. To correct for batch effects, tissue origin (“Lung-CAF”, “LN-CAF”, and “NF”) was included as a covariate in the edgeR design matrix for differential expression analysis. Significant DE genes were selected based on the following criteria: FDR-adjusted *p*-value < 0.05, logCPM > 2, and absolute log_2_ fold change > 1. K-means-based clustering was performed on expression profiles of significant DE genes, and heatmaps were generated using the R package ComplexHeatmap v2.10.0 (https://bioconductor.org/packages/release/bioc/html/ComplexHeatmap.html [accessed on 30 August 2025]) [23]. Gene set enrichment analysis was performed with HALLMARK, C2 (curated), and C6 (oncogenic) gene sets including KEGG pathways [24] from the R package MSigDB v7.4.1 (https://www.gsea-msigdb.org/gsea/msigdb [accessed on 30 August 2025]) [25], based on GSVA scores using the R package GSVA v1.42.0 (https://bioconductor.org/packages/release/bioc/html/GSVA.html [accessed on 30 August 2025]) [26]. Gene sets were considered to exhibit significant DE based on the following criteria: absolute log_2_ fold change > 0.4 and FDR-adjusted *p*-value < 0.05 by a non-parametric (e.g., Kruskal–Wallis) test. For pairwise comparison, *t*-tests were performed, and *p*-values were FDR-adjusted.

### 2.7. Prediction of CAF Subpopulation

Fibroblast identity was further confirmed by RNA-seq analysis showing a robust expression of positive fibroblast markers including FAP, ACTA2, PDGFRA, and PDGFRB (all logCPM > 12) in both CAF and NF populations. To decipher CAFs into three subpopulations (myCAFs, iCAFs, and apCAFs), GSVA scores using the GSVA R package (v1.42.0) [26] were estimated on the basis of specific gene sets reported in pancreatic cancer [27]. The myCAFs with lung cancer annotations were also estimated with GSVA scores according to highly expressed genes (*COL4A1*, *ACTA2*, *MEF2C*, *MYG11*, and *ITGA7*) [12]. Differences in GSVA scores among CAF subpopulations were examined using the Kruskal–Wallis test; the threshold for statistical significance was the FDR-adjusted *p*-value < 0.05. All statistical analyses were performed using R version 4.1.2 (https://www.r-project.org).

### 2.8. Western Blotting

Cells were harvested and suspended in lysis buffer (150 mM NaCl, 25 mM Tris–Cl, pH 7.4, 1% NP-40, and 10 mM EDTA) containing a protease inhibitor cocktail (Roche, Mannheim, Germany). After boiling for 10 min, the cell lysate was centrifuged at 13,000 rpm for 10 min at room temperature (RT). Protein samples were estimated using a modified BCA protein assay reagent (Pierce, Rockford, IL, USA, cat. no. 23228). A total of 20 µg volumes of protein samples were electrophoresed on 10% sodium dodecyl-sulfate polyacrylamide gel electrophoresis (SDS-PAGE) gradient gels and transferred onto PVDF membrane (Bio-Rad, Hercules, CA, USA) using the Trans-Blot electrophoretic cell transfer system (Bio-Rad Laboratories, Hercules, CA, USA). Protein-transferred PVDF membranes were incubated for 1 h in 5% BSA in 1× TBST (150 mM NaCl, 50 mM Tris–Cl, pH 7.5, and 0.1% Tween 20), washed with 1× TBST, and then incubated overnight at 4 °C in a primary antibody appropriately diluted in 5% BSA in TBST. Detection was performed with a chemiluminescent substrate (Thermo Fisher), followed by exposure to an ECL solution (Thermo Fisher, Waltham, MA, USA, 34094). Western blotting was performed by applying primary antibodies for GAS6 (Abcam, Cambridge, Cambridgeshire, UK, ab264098), GREM1 (R&D Systems, Minneapolis, MN, USA, AF956), α-SMA (Abcam, ab7817), and β-actin (Santa Cruz, Dallas, TX, USA, sc-47778) to the blot in ratios of 1:500. Secondary antibodies used were anti-Rabbit IgG H&L (Abcam, ab97051), anti-goat IgG (Santa Cruz Biotechnology, Dallas, TX, USA, sc-2354), and anti-mouse IgG (Santa Cruz Biotechnology, sc-516102), each applied at a 1:5000 dilution. Quantification of Western blot signals was performed using the replicate samples as indicated. For Western blot quantification, we used ImageJ 1.54g software. Densitometry analysis was performed by comparing the expression of β-actin, which was used as the standard blot for normalization. The results were presented as the ratio of the normalized signal of the sample to the control.

### 2.9. THP-1 Cell Culture and Differentiation

THP-1 cells (Korean Cell Line Bank, Seoul, KR, #40202) were maintained in RPMI 1640 medium (Gibco, #11875093) supplemented with 10% FBS (Gibco, #16000044), 1% Penicillin-Streptomycin (Gibco, #15140-122), and 50 μM 2-mercaptoethanol (Sigma, #M-3148) at 37 °C in a humidified 5% CO_2_ incubator. For macrophage differentiation, cells were treated with 320 nM PMA (Sigma, #P1585) for 24 h, followed by a 24 h rest in PMA-free medium.

### 2.10. Co-Culture with CAFs and siRNA-Mediated KNOCKDOWN

CAFs were detached using Trypsin-EDTA, counted, and co-cultured with differentiated M0 macrophages at a 5:1 ratio (M0:CAF) in a 1:1 mixture of M0 and CAF media. For indirect co-culture, trans-well inserts (0.4 μm pore size; Costar, Corning, NY, USA, #3470) were used, with CAFs seeded in the upper chamber and M0 macrophages in the lower chamber. Co-culture was maintained for 72 h.

For gene knockdown, CAFs were transfected with 10 μM siRNAs targeting GAS6 (Santa Cruz, #sc-35450; 20 μL) or GREM1 (Santa Cruz, #sc-35408; 10 μL) using TransIT transfection reagent (Mirus, Madison, WI, USA) 6 h after seeding 2 × 10^6^ cells/flasks. After 72 h incubation, siRNA-treated CAFs were collected using Trypsin-EDTA and used in trans-well co-cultures with M0 macrophages, as described above.

### 2.11. Real-Time Quantitative PCR (qPCR)

Total RNA was extracted from macrophages using TRIzol reagent (Invitrogen, #15596026) and quantified with a NanoDrop 2000 spectrophotometer (Thermo Scientific). cDNA was synthesized from 1 µg of total RNA using AccuPower^®^ CycleScript RT Premix (dT20) (Bioneer, Daejeon, KR, #K-2044).

qPCR was performed on a QuantStudio™ 6 Flex Real-Time PCR System (Thermo Fisher Scientific, 384-well) using a SYBR Green PCR Master Mix (Applied Biosystems, #4367659). Each 10 μL reaction contained 4 μL of 2× SYBR Green mix, 2 μL of 10 pmol forward and reverse primers, and 4 μL of cDNA. Cycling conditions were: 95 °C for 10 min, followed by 40 cycles of 95 °C for 15 s and 60 °C for 1 min. Melting curve analysis was performed from 60 °C to 95 °C, increasing at 0.05 °C/s. All reactions were conducted in triplicate.

Relative gene expression was calculated using the 2^−ΔΔCt^ method, with GAPDH as the internal control. Statistical analysis between M0 and other groups was performed using an unpaired *t*-test (* *p* < 0.05). Primer sequences are listed in Table S1.

## 3. Results

### 3.1. Study Population

**Figure 1:**
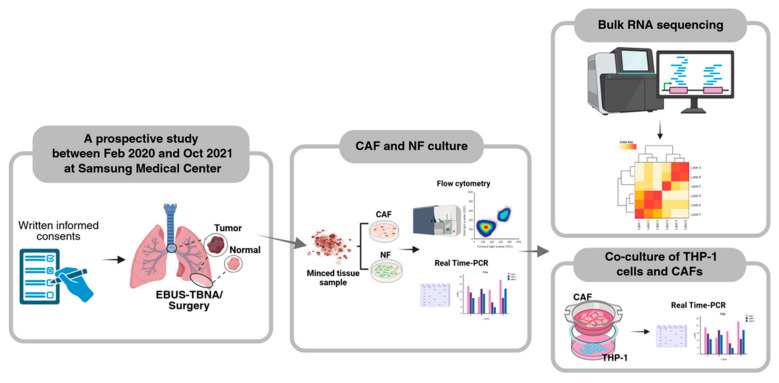
Summary of the study design. CAF, cancer-associated fibroblast; NF, normal fibroblast; and EBUS-TBNA, endobronchial ultrasound-guided transbronchial needle aspiration.

Between February 2020 and October 2021, 27 CAFs and 12 NFs were isolated and cultured from tumor and non-tumor tissues in patients with treatment-naïve NSCLC (Data S1). Among them, five CAFs and one NF were excluded from RNA sequencing or functional analyses due to epithelial cell contamination (N = 2) or failure of further passage (N = 4). Consequently, 22 CAFs and 11 NFs were included in the RNA sequencing analysis (Data S1). Three CAFs (TH122, TH127, and TH128) were subjected to both RNA sequencing and functional analyses, while one CAF (TH179) was included only in the functional analysis (Data S1). The 22 CAF samples comprised 12 adenocarcinomas and 10 squamous cell carcinomas (SqCCs): 16 from primary tumors and 6 from metastatic lymph nodes via EBUS-TBNA. Additionally, 12 samples were from stage I or II cancers, and 10 samples were from stage III or IV cancers. The 11 NF samples included 8 adenocarcinomas and 3 SqCCs, all derived from non-tumorous lungs (Figure 1). Ten pairs of CAFs and NFs were isolated from the same patients (Data S1). Table 1 presents a summary of all patient characteristics.

To validate the purity of the isolated CAFs and NFs, we assessed the expression of epithelial markers by RT-PCR and flow cytometry. Specifically, the absence of E-cadherin expression in RT-PCR and an EpCAM expression level below 1% in FACS were confirmed prior to subsequent experiments (Figure S1).

### 3.2. Differentially Expressed Genes (DEGs) Between CAFs and NFs

Fibroblast identity and purity was also confirmed by the robust expression of FAP, ACTA2, PDGFRA, and PDGFRB in both CAFs and NFs (logCPM > 12), confirming the fibroblast identity of our isolated cell populations (Figure S2).

**Figure 2:**
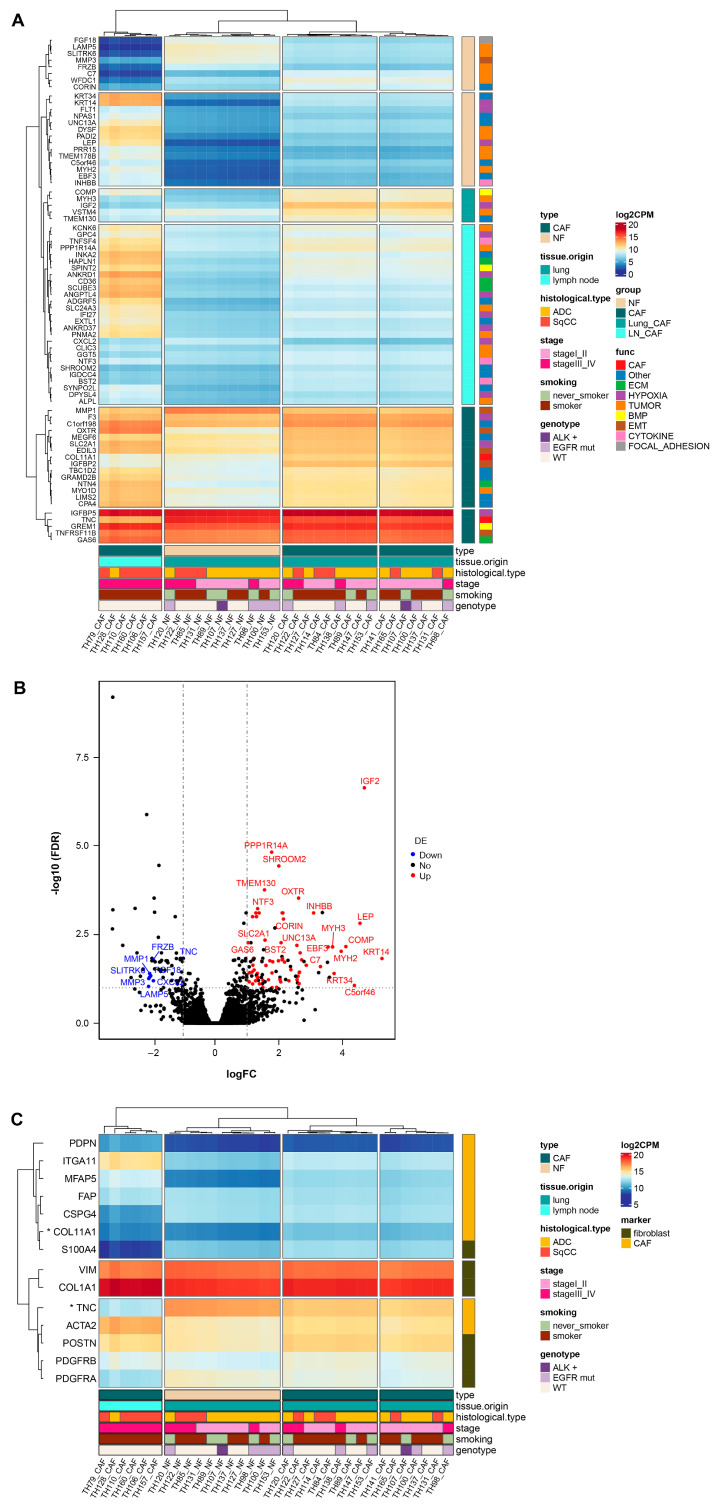
DEGs between CAFs and NFs. (**A**) Heatmap of 74 DEGs, which distinctly separated CAFs from NFs, as well as distinguishing LN-CAFs from Lung-CAFs through unsupervised clustering. (**B**) Volcano plot revealed 66 genes (e.g., GAS6, GREM1, CD36, COL11A1, and IGF2) significantly upregulated in CAFs, whereas 8 genes (e.g., TNC, CXCL2, and MMP1) were downregulated. (**C**) Heatmap of known fibroblast marker genes, which also distinctly separated CAFs from NFs and LN-CAFs from Lung-CAFs. * Significant DEG. CAF, cancer-associated fibroblast; NF, normal fibroblast.

Figure 2 shows the DEGs between CAFs and NFs using 22 CAF samples and 11 NF samples. Figure 2A shows a heatmap of 74 DEGs, which distinctly separated CAFs from NFs as well as distinguishing LN-CAFs from Lung-CAFs through unsupervised clustering. Volcano plots revealed 66 genes (e.g., GAS6, GREM1, CD36, COL11A1, and IGF2) significantly upregulated in CAFs, whereas 8 genes (e.g., TNC, CXCL2, and MMP1) were downregulated (Figure 2B, Data S2). Figure 2C shows a heatmap of significant DEGs from known fibroblast markers [28], which also distinctly separated CAFs from NFs and LN-CAFs from Lung-CAFs.

**Figure 3:**
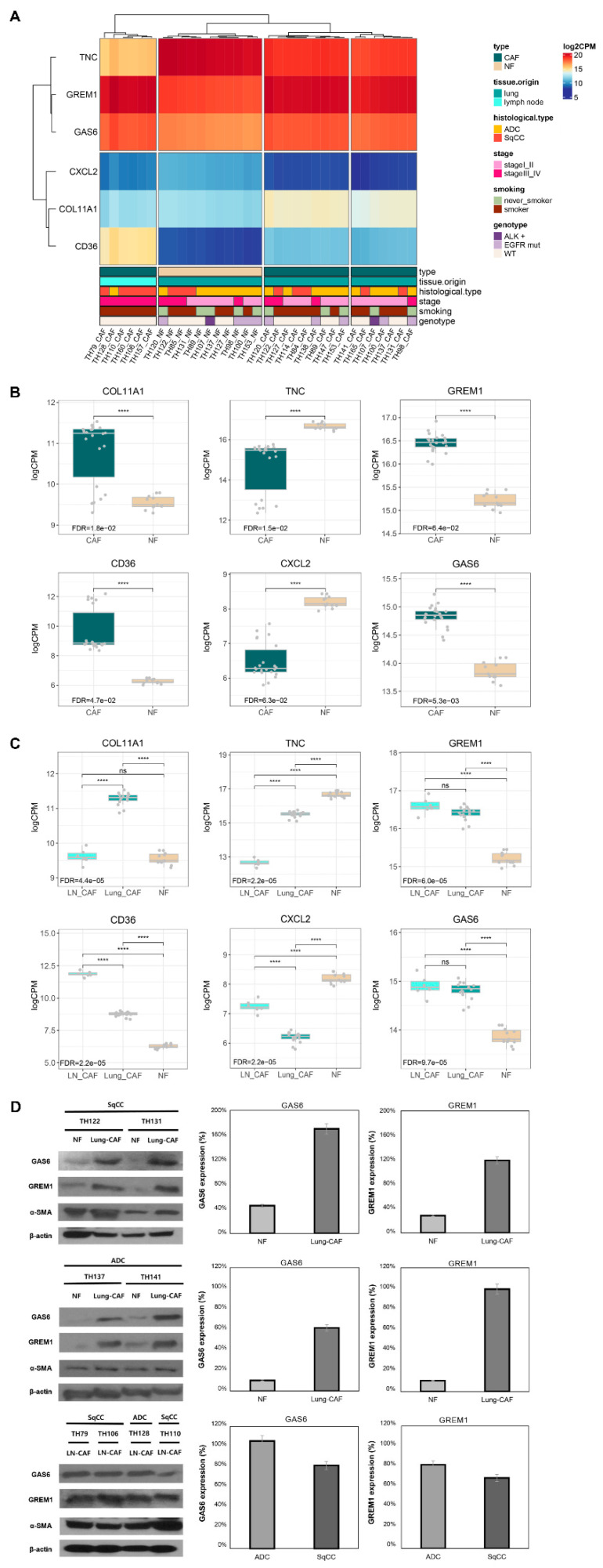
Six selected DEGs between CAFs and NFs. (**A**) Heatmap of six selected DEGs between CAFs and NFs. (**B**) Boxplot of DEGs comparing CAFs and NFs. Adjusted *p*-values (FDR) from generalized linear model. COL11A1 and TNC, known fibroblast markers; GREM1, BMP pathway; CD36, antigen processing machinery pathway; CXCL2, chemokine pathway; and GAS6, hypoxia pathway. (**C**) Boxplot of DEGs comparing LN-CAFs, Lung-CAFs, and NFs. (**D**) Western blot analysis (**left**) and densitometry-based quantification (**right**) of GREM-1 and GAS6 in LN-CAFs, Lung-CAFs, and NFs derived from squamous cell carcinomas, adenocarcinomas, and non-tumorous lungs. Adjusted *p*-values (FDR): ns, *p* > 0.05; ****, and *p* ≤ 0.0001. CAF, cancer-associated fibroblast; NF, normal fibroblast; SqCC, squamous cell carcinoma; and ADC, adenocarcinoma.

Among 74 DEGs, we identified six important genes to differentiate CAFs and NFs: *COL11A1* and *TNC* (fibroblast markers), *GREM1* (bone morphogenetic protein [BMP] pathway), *CD36* (antigen processing machinery pathway), *CXCL2* (chemokine pathway), and *GAS6* (hypoxia pathway). Figure 3 shows the DEGs of these six genes between CAFs and NFs. Figure 3A shows a heatmap of these DEGs, clearly differentiating CAFs from NFs and LN-CAFs from Lung-CAFs. *COL11A1*, *GREM1*, *CD36*, and *GAS6* were substantially higher in CAFs, whereas *TNC* and *CXCL2* showed elevated levels in NFs (all with a false discovery rate [FDR], *p* ≤ 0.0001) (Figure 3B). *COL11A1* expression was higher in Lung-CAFs than in LN-CAFs or NFs (FDR, *p* ≤ 0.0001) (Figure 3C). *CD36* expression was greatest in LN-CAFs, surpassing the levels in Lung-CAFs and NFs (all FDR, *p* ≤ 0.0001). *CXCL2* expression was higher in LN-CAFs than in Lung-CAFs, but it remained lower than the expression in NFs (all FDR, *p* ≤ 0.0001). Both *GREM1* and *GAS6* showed a robust expression in Lung-CAFs and LN-CAFs relative to NFs (all FDR, *p* ≤ 0.0001) (Figure 3C). The average expression levels of GREM1, GAS6, TNC, CD36, CXCL12, and COL11A1 in CAFs and NFs are summarized in Table S3.

A Western blot analysis also showed the higher protein expression of GREM1 and GAS6 in both LN-CAFs and Lung-CAFs compared with NFs, regardless of histological subtypes (Figure 3D and Data S4).

### 3.3. Gene Set Enrichment Analysis Between CAFs and NFs

**Figure 4:**
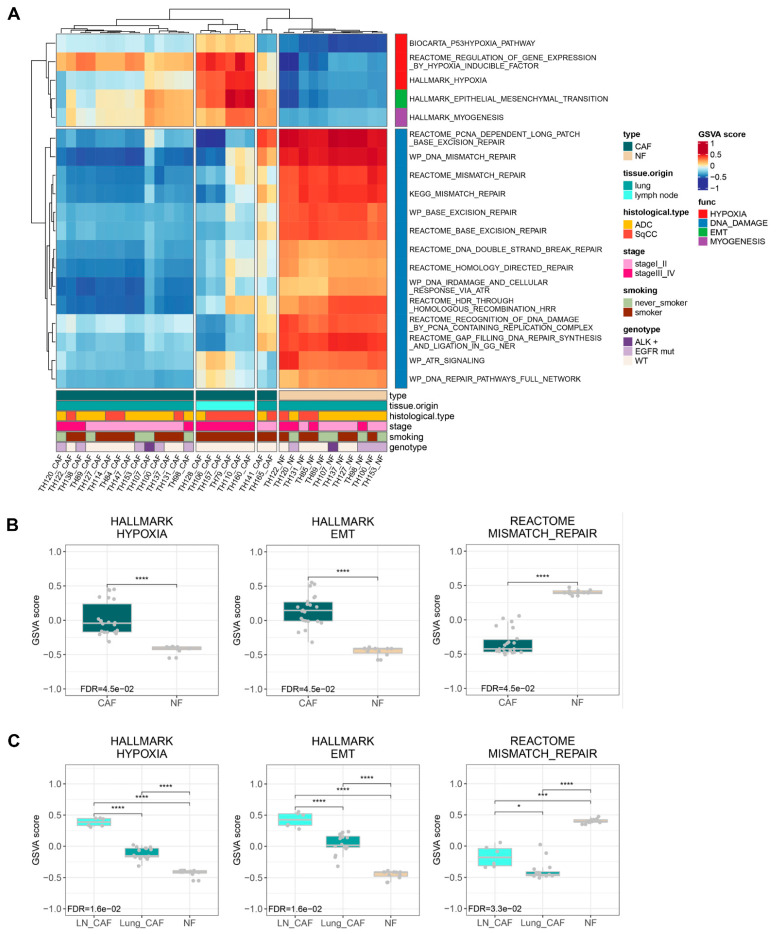
DEG sets between CAFs and NFs. (**A**) Heatmap of GSVA scores in selected DEG sets among well-known pathway databases (e.g., HALLMARK, BIOCARTA, KEGG, REACTOME, and WIKI pathway). (**B**) Boxplot of GSVA scores in selected DEG sets comparing CAFs and NFs. Gene sets related to hypoxia responses (Hallmark Hypoxia) and EMT (Hallmark EMT) were upregulated in CAFs compared with NFs (all FDR, *p* ≤ 0.0001). Conversely, the gene set related to mismatch repair (Reactome Mismatch Repair) exhibited significantly higher GSVA scores in NFs than in CAFs (FDR, *p* ≤ 0.0001). (**C**) Boxplot of GSVA scores in selected DEG sets comparing LN-CAFs, Lung-CAFs, and NFs. LN-CAFs showed higher GSVA scores for gene sets such as Hallmark Hypoxia and Hallmark EMT, compared with Lung-CAFs (FDR, *p* ≤ 0.0001). Adjusted *p*-values (FDR): *, *p* ≤ 0.05; ***, *p* ≤ 0.001; ****, and *p* ≤ 0.0001. CAF, cancer-associated fibroblast; EMT, epithelial–mesenchymal transition; NF, normal fibroblast; and GSVA, gene set variation analysis.

Figure 4A shows a heatmap of gene set variation analysis (GSVA) scores for selected gene sets (Data S3) comparing CAFs with NFs. GSVA scores clearly differentiated CAFs from NFs but did not completely distinguish LN-CAFs from Lung-CAFs. Gene sets related to hypoxia responses (Hallmark Hypoxia) and epithelial–mesenchymal transitions (EMT) (Hallmark EMT) were upregulated in CAFs compared with NFs (all FDR, *p* ≤ 0.0001). Conversely, the gene set related to mismatch repair (Reactome Mismatch Repair) exhibited significantly higher GSVA scores in NFs than in CAFs (FDR, *p* ≤ 0.0001) (Figure 4B). Additionally, LN-CAFs showed higher GSVA scores for gene sets such as Hallmark Hypoxia and Hallmark EMT compared with Lung-CAFs (FDR, *p* ≤ 0.0001) (Figure 4C).

### 3.4. CAF Subpopulation

**Figure 5:**
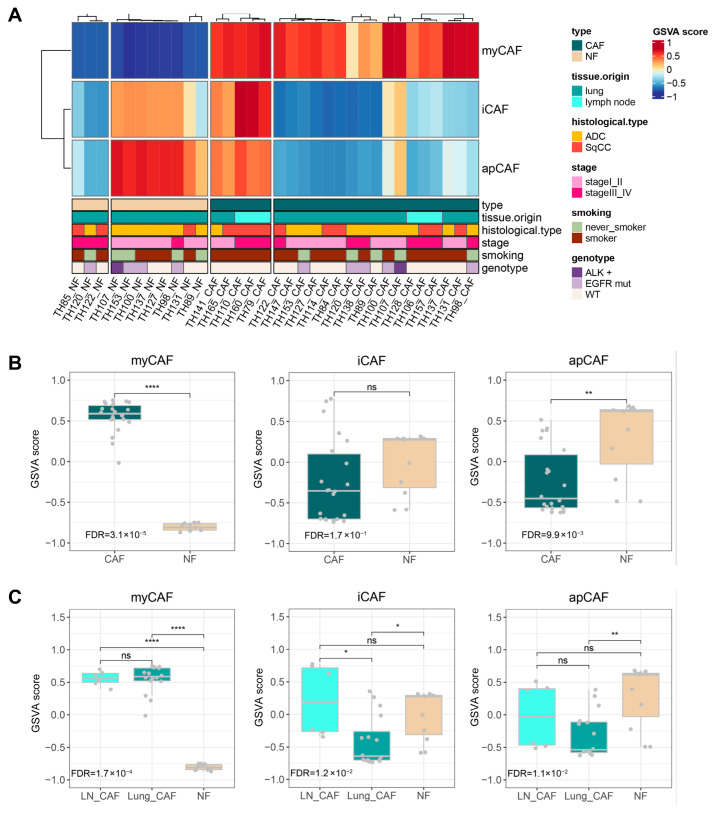
CAF subpopulation prediction. (**A**) Heatmap of CAF subpopulation based on GSVA scores. (**B**) Boxplot of GSVA scores for CAF subpopulations comparing CAFs and NFs. CAFs had a significantly higher GSVA score for the myCAF subpopulation compared with NFs (FDR, *p* ≤ 0.0001), whereas NFs exhibited significantly higher GSVA scores for the apCAF subpopulation relative to CAFs (FDR, *p* ≤ 0.01). GSVA scores for the iCAF subpopulation were similar between CAFs and NFs. (**C**) Boxplot of GSVA scores for CAF subpopulations comparing LN-CAFs, Lung-CAFs, and NFs. Both Lung-CAFs and LN-CAFs showed significantly higher GSVA scores for the myCAF subpopulation compared with NFs (all FDR, *p* ≤ 0.0001), whereas NFs had significantly higher GSVA scores for the apCAF subpopulation relative to Lung-CAFs (FDR, *p* ≤ 0.01). Additionally, LN-CAFs and NFs had significantly higher GSVA scores for the iCAF subpopulation compared with Lung-CAFs (all FDR, *p* ≤ 0.05). Adjusted *p*-values (FDR): ns, *p* > 0.05; *, *p* ≤ 0.05; **, *p* ≤ 0.01; ****, and *p* ≤ 0.0001. CAF, cancer-associated fibroblast; NF, normal fibroblast; and GSVA, gene set variation analysis.

Figure 5A shows a heatmap of subpopulations for CAFs and NFs based on previously reported gene sets [27], which clearly differentiated CAFs from NFs but did not completely differentiate LN-CAFs from Lung-CAFs. CAFs had a significantly higher GSVA score for the myofibroblast CAF (myCAF) subpopulation compared with NFs (FDR, *p* ≤ 0.0001), whereas NFs exhibited significantly higher GSVA scores for the antigen-presenting CAF (apCAF) subpopulation relative to CAFs (FDR, *p* ≤ 0.01). GSVA scores for the inflammatory CAF (iCAF) subpopulation were similar between CAFs and NFs (Figure 5B). Both Lung-CAFs and LN-CAFs showed significantly higher GSVA scores for the myCAF subpopulation compared with NFs (all FDR, *p* ≤ 0.0001), whereas NFs had significantly higher GSVA scores for the apCAF subpopulation relative to Lung-CAFs (FDR, *p* ≤ 0.01). Additionally, LN-CAFs and NFs had significantly higher GSVA scores for the iCAF subpopulation compared with Lung-CAFs (all FDR, *p* ≤ 0.05) (Figure 5C).

### 3.5. CAF-Derived Signals Promote M2-like Polarization of Macrophages via GREM1 and GAS6

**Figure 6:**
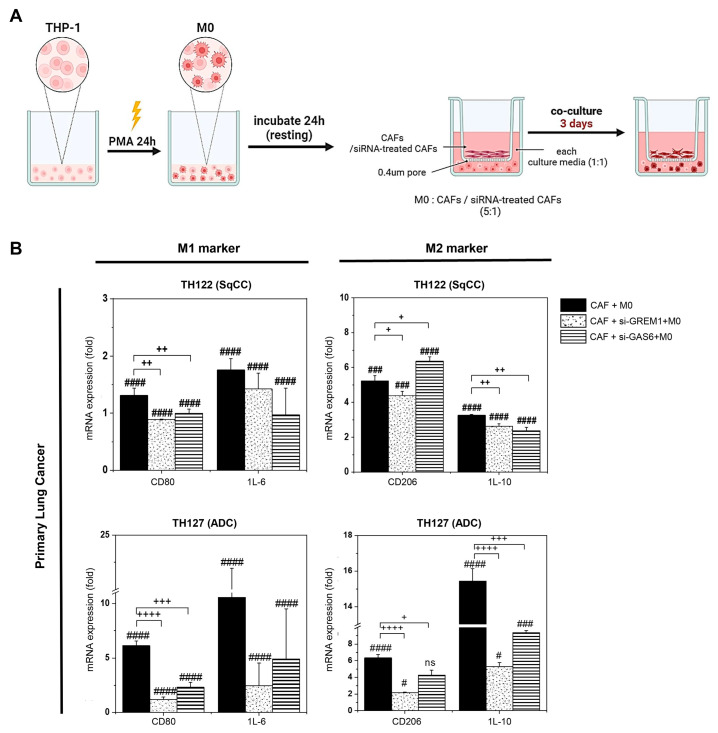
M2 polarization of macrophages by CAFs and its reversal via *GAS6* or *GREM1* knockdown. (**A**) Schematic representation of the co-culture system. THP-1 monocytes were differentiated into M0 macrophages using PMA treatment for 24 h, followed by 24 h of resting. M0 macrophages were then co-cultured with CAFs or siRNA-treated CAFs in a 0.4 μm pore size trans-well system for 3 days at a ratio of 5:1 (M0 macrophages: CAFs) in a mixed medium (1:1 ratio of M0 macrophage and CAF medium). (**B**) RT-qPCR analysis of M1 (CD80, IL-6) and M2 (CD206, IL-10) macrophage marker expressions after 3-day co-culture with CAFs derived from primary lung tumors (TH122, TH127). Co-culture with CAFs significantly induced both M1 and M2 markers compared to untreated M0 macrophages. Knockdown of GAS6 or GREM1 in CAFs using siRNA significantly reduced M1/M2 marker expression in M0 macrophages. ADC; adenocarcinoma, SqCC; squamous cell carcinoma. (# *p* < 0.05, ### *p* < 0.001, and #### *p* < 0.0001 vs. M1 or M2; + *p* < 0.05, ++ *p* < 0.01, +++ *p* < 0.001, and ++++ *p* < 0.0001 vs. CAF co-culture; and ns = not significant).

To assess the influence of CAFs on macrophage polarization, we performed co-culture experiments using PMA-induced M0 macrophages and CAFs derived from NSCLC patients. As illustrated in Figure 6A, M0 macrophages were co-cultured with CAFs or CAFs transfected with siRNAs targeting *GREM1* or *GAS6* using a trans-well system for 3 days.

RT-qPCR analysis revealed that co-culture with CAFs led to a marked upregulation of both M1 (CD80 and IL-6) and M2 (CD206 and IL-10) macrophage markers across all CAF samples (Figure 6B). Notably, the knockdown of *GREM1* or *GAS6* in CAFs significantly reduced the expression of M2 markers (CD206 and IL-10), whereas the reduction in M1 markers was less consistent. This suggests that CAF-derived *GREM1* and *GAS6* primarily contribute to skewing macrophages toward an M2 phenotype within the tumor microenvironment (TME).

## 4. Discussion

We investigated CAF-specific markers through the whole-transcriptome analysis of 22 CAFs and 11 NFs from patients with NSCLC. Notably, *COL11A1*, *GREM1*, *CD36*, and *GAS6* were highly expressed in CAFs, whereas *TNC* and *CXCL2* were elevated in NFs. Specifically, *CD36* expression in LN-CAFs exceeded that in Lung-CAFs and NFs. Additionally, the CXCL2 expression was higher in LN-CAFs than in Lung-CAFs, although it remained lower than the expression in NFs. Similarly, *COL11A1* expression in Lung-CAFs surpassed that in LN-CAFs and NFs; *TNC* expression was higher in Lung-CAFs than in LN-CAFs but remained lower than in NFs. Both *GREM1* and *GAS6* showed a robust expression in Lung-CAFs and LN-CAFs compared with NFs. Moreover, Lung-CAFs and LN-CAFs showed a higher protein expression of GREM1 and GAS6 compared with NFs. Although the samples in our study included both adenocarcinomas and SqCCs, they did not cluster by histologic types in the heatmap.

CAFs constitute a key component of the TME and play crucial roles in tumor maintenance and progression through various mechanisms [29]. These mechanisms include promoting tumor cell growth by releasing growth factors, chemokines, and extracellular vesicles, as well as ECM remodeling. Consequently, CAFs regulate key aspects of tumor behavior, such as proliferation, metastasis, therapeutic resistance, immune evasion, and the recently discovered reactivation from tumor dormancy [30,31]. Research concerning CAFs in lung cancer continues to advance, but the molecular heterogeneity of CAFs presents scientific and technical challenges that require further investigation. Specifically, the relationship and function of CAFs at metastatic and primary sites remain unresolved. In our study, we analyzed CAFs from the primary cancer site (lung) and separately examined CAFs from metastatic lymph nodes. This approach allowed us to identify genes highly expressed in each specimen type. Whereas previous studies primarily focused on CAFs in primary lung cancer, our research contributes unique insights into CAFs in metastatic lymph nodes, revealing differences in gene expression profiles between Lung-CAFs and LN-CAFs in NSCLC.

*CD36*, a transmembrane glycoprotein, interacts with various ligands including fatty acids, cholesterol, thrombospondin-1, and thrombospondin-2. It plays a crucial role in lipid metabolism, immune response, and angiogenesis [32]. Recent studies have highlighted the role of *CD36* in mediating lipid uptake by tumor-associated immune cells and promoting tumor cell progression [33]. Within CAFs, *CD36* regulates lipid uptake and matrix protein production, contributing to tumor proliferation and angiogenesis through vascular mimicry [32,33]. Our analysis showed that *CD36* expression was higher in LN-CAFs than in Lung-CAFs in the context of NSCLC.

*COL11A1* is a recognized cancer-specific fibroblast marker that can promote tumor progression by influencing ECM remodeling and anti-tumor immune responses [34,35]. In our study, *COL11A1* expression was higher in CAFs than in NFs; it was substantially higher in Lung-CAFs than in LN-CAFs.

Lung-CAFs and LN-CAFs exhibit distinct gene expression patterns, suggesting functional adaptations based on tumor location. *CD36*, highly expressed in LN-CAFs, may support lipid metabolism-driven tumor survival in metastatic lymph nodes, whereas *COL11A1*, elevated in Lung-CAFs, likely enhances ECM remodeling at the primary tumor site. These findings may highlight potential therapeutic targets based on tumor location—*CD36* inhibition could impede metastatic progression, while a *COL11A1* blockade may disrupt primary tumor development.

Both *GREM1* and *GAS6* emerged as highly enriched genes in CAFs compared with NFs, regardless of whether they originated from primary tumors (Lung-CAFs) or metastatic lymph nodes (LN-CAFs). *GREM1*, an antagonist of BMPs, is expressed within CAFs both in vitro and in vivo [36]. *GREM1* disrupts BMP/SMAD signaling in breast cancer cells, promoting their mesenchymal phenotype, stemness, and invasion [36]. Furthermore, *GREM1* is highly expressed within NSCLC tissue and acts as a proto-oncogene, contributing to disease development and progression [37]. Although the specific role of *GREM1* in NSCLC CAFs requires further investigation, our findings indicate that *GREM1* is highly expressed in Lung-CAFs and LN-CAFs. *GAS6*, secreted by CAFs, facilitates the migration of Axl-expressing lung cancer cells during chemotherapy [38,39]. Kanzaki et al. found that *GAS6* expression in CAFs was upregulated after cisplatin treatment [38]. *GAS6* levels may be affected by intratumoral hypoperfusion during chemotherapy; they can increase after serum starvation in human Lung-CAFs [38]. Recombinant *GAS6* promotes lung cancer cell migration, and its expression in the tumor stroma is correlated with poor clinical outcomes. The GAS6/Axl pathway is implicated in resistance to tumor therapy [39]. However, one recent study using mouse models has shown that Gas6 signaling can alleviate pulmonary fibrosis by inhibiting epithelial–mesenchymal transition and fibroblast activation [40]. In our study, *GAS6* was significantly overexpressed in CAFs compared to NFs, suggesting a tumor-promoting role in the TME. While Gas6 may exert anti-fibrotic effects by regulating epithelial and immune cell responses in the context of acute lung injury or idiopathic pulmonary fibrosis, its function within the TME is likely to differ. Further studies are needed to confirm this.

*GREM1* and *GAS6* could serve as valuable biomarkers for NSCLC-derived CAFs and potential therapeutic targets in NSCLC. To our knowledge, this is the first study to identify *GREM1* and *GAS6* as specific biomarkers for Lung-CAFs and LN-CAFs in NSCLC. Further research is needed to explore the therapeutic implications of *GREM1* and *GAS6* in NSCLC.

Because CAFs are highly heterogeneous cells with distinct gene expression patterns and sometimes opposite biological functions within the TME, distinct CAF subpopulations can exist within a single tumor [27]. Several studies using single-cell RNA sequencing and genetically engineered mouse models have begun to reveal the heterogeneity and functional roles of CAFs, which are dynamic and context-dependent. myCAFs exhibit a myofibroblastic phenotype, characterized by the expression of *α-SMA*, and they play a role in tissue remodeling and matrix deposition. Their hyperproliferative nature distinguishes them from other CAF populations [41]. iCAFs, characterized by the expression of cytokines such as IL-6 and CXCL12, contribute to the inflammatory milieu within the TME [42]. apCAFs express major histocompatibility complex II family genes [42], potentially influencing immune responses, and may be colocalized with lymphoid immune cells in early tumors [43].

In our study, myCAF was elevated in both Lung-CAFs and LN-CAFs, iCAF was elevated in LN-CAFs and NFs, and apCAF was elevated in NFs relative to Lung-CAFs. Our findings highlight distinct CAF subpopulation compositions in Lung-CAFs, LN-CAFs, and NFs. Based on our findings, the predominance of myCAF traits in both Lung-CAFs and LN-CAFs suggests a conserved, tumor-promoting activation state likely driven by TGF-β signaling or mechanical stress within the NSCLC microenvironment [44]. These myCAFs may contribute to immunosuppression and tumor progression through extracellular matrix remodeling [45]. In contrast, the elevated apCAF features in normal fibroblasts could reflect tissue-specific properties or be influenced by in vitro culture conditions [46]. Further studies using freshly isolated cells are needed to determine whether these traits represent stable subsets or culture-induced artifacts.

Classifications of CAFs across various tumor types generally converge on three main subtypes: myCAF, iCAF, and apCAF [27,47]. Although previous studies have reported unique CAF subtypes in lung cancer identified through scRNA sequencing (clusters 1, 2, 4, 5, and 7) [12] and therapeutic profiling (subtypes 1, 2, and 3) [48], these subtypes have not been consistently reproduced by other researchers. Although we used pancreatic CAF subpopulation signatures for NSCLC in our study, further research is needed to identify the optimal subpopulations and their specific markers in NSCLC.

In this study, the co-culture of CAFs and M0 macrophages resulted in a marked upregulation of both M1 and M2 macrophage markers. However, the knockdown of *GREM1* or *GAS6* in CAFs significantly reduced the expression of M2 markers, while having minimal impact on M1 markers. These findings suggest that *GREM1* and *GAS6* are critical for maintaining the M2 phenotype of tumor-associated macrophages (TAMs). TAMs play an essential role in sustaining an immunosuppressive TME [49]. In a previous study, a high density of M1 TAMs within the tumor islet was associated with improved overall survival (OS), whereas a high density of M2 TAMs in the tumor stroma correlated with a poorer OS [50]. Therefore, targeting *GREM1* or *GAS6* may represent a promising strategy to mitigate immunosuppressive TAM phenotypes. These findings also suggest that targeting *GREM1* and *GAS6* might offer a therapeutic strategy to reprogram the TME and enhance anti-tumor immunity. Future in vivo studies will be essential to validate these effects and assess their impact on tumor progression.

The present study investigated differences between CAFs from primary tumors, metastatic lymph nodes, and NFs from non-tumorous lungs in patients with NSCLC. We identified key genes associated with CAFs and observed distinct characteristics in each tissue type. However, our findings require validation in additional studies, and further investigation is needed to understand the impact of these differences on treatment outcomes or treatment responses. Although *GREM1* and *GAS6* were consistently upregulated in CAFs compared to NFs at both the transcriptomic and protein levels, their expression may not be exclusive to CAFs and could be present in other cell types within the TME. To clarify their cellular origin and assess their potential as reliable CAF-enriched markers in NSCLC, further validation using single-cell RNA sequencing or spatial transcriptomics is warranted.

In this study, fibroblasts derived from tumor tissues were classified as CAFs, while those derived from non-tumor tissues were classified as NFs. Owing to the heterogeneous nature and high plasticity of fibroblasts, those isolated from tumor tissues cannot be uniformly regarded as CAFs [28]. However, our recent study demonstrated that CAFs derived from tumor tissues promoted immune evasion by suppressing the function of CD4^+^ and CD8^+^ T cells [19]. Furthermore, in a co-culture model of patient-matched tumor organoids and CAFs, PDTOs co-cultured with CAFs exhibited an increased resistance to paclitaxel chemotherapy compared with PDTOs cultured alone [51]. Future studies are required to better define the identity and function of CAFs and NFs.

## 5. Conclusions

In conclusion, *GREM1* and *GAS6* were identified as specific markers for Lung-CAFs and LN-CAFs in NSCLC. *CD36* and *COL11A1* were prominently expressed in LN-CAFs and Lung-CAFs, respectively. *CXCL2* and *TNC* were specific markers for NFs. CAFs exhibited characteristics of the myCAF subpopulation, whereas NFs showed characteristics of the apCAF subpopulation. *GREM1* and *GAS6* could serve as valuable diagnostic targets for CAFs derived from NSCLC; this possibility requires further exploration.

## Figures and Tables

**Table 1:** Short summary table with patient characteristics.

Variables	Total Patients (n = 29)
Age, years	63 (59–69)
Sex	
Male	22 (75.9)
Female	7 (24.1)
Smoking history	
Never-smoker	6 (20.7)
Smoker	23 (79.3)
Stage *	
Stage I–II	13 (44.8)
Stage III–IV	16 (55.2)

Data are presented as median (interquartile range) or number (percentage). * Based on the TNM eighth edition.

## Data Availability

Data reported in this work will be made available from the lead contact upon request to the corresponding author. The code for analysis in this work will be made available from the lead contact upon request to the corresponding author. Any additional information required to reanalyze the data reported in this work is available from the lead contact upon request to the corresponding author. The datasets generated and/or analyzed during the current study are available in the Gene Expression Omnibus repository (GEO), GSE300983. To review GEO accession GSE300983: go to https://www.ncbi.nlm.nih.gov/geo/query/acc.cgi?acc=GSE300983. Enter token glexiqomdzqvrkr into the box.

## Supplementary Materials

The following supporting information can be downloaded at: https://www.mdpi.com/article/10.3390/cancers17172858/s1, Data S1: Excel file containing the baseline characteristics of study population; Data S2: Excel file containing the DEGs between CAFs and NFs; Data S3: Excel file containing the GSVA scores in selected gene sets comparing CAFs with NFs; Data S4: Original version of whole Western blot for Figure 3D; Figure S1: Representative results of RT-PCR (A) and flow cytometry (B) for the evaluation of the purity of CAFs and NFs; Figure S2: The expression of canonical positive fibroblast markers; Table S1: List of primer sequences used for real-time qPCR; Table S2: List of antibodies used in this study; and Table S3: Average expression values for CAFs and NFs.

## Abbreviations

αSMA	α-smooth muscle actin
apCAF	antigen-presenting CAF
CAF	cancer-associated fibroblast
DE	differential expression
DEG	differentially expressed gene
ECM	extracellular matrix
FAP	fibroblast-associated protein
FDR	false discovery rate
GSVA	gene set variation analysis
iCAF	inflammatory CAF
Lung-CAFs	lung-origin CAFs
LN-CAFs	lymph node-origin CAFs
myCAF	myofibroblast CAF
NF	normal fibroblast
NSCLC	non-small cell lung cancer
SqCC	squamous cell carcinoma
