# Correction: Kim et al. Identification of *GREM-1* and *GAS6* as Specific Biomarkers for Cancer-Associated Fibroblasts Derived from Patients with Non-Small-Cell Lung Cancer. *Cancers* 2025, *17*, 2858

**DOI:** 10.3390/cancers18111853

**Published:** 2026-06-05

**Authors:** Bo-Guen Kim, Kyunghee Park, Mina Hwang, Hyewon Lee, Kyung-Mi Park, Junsu Choe, Sun Hye Shin, Byeong-Ho Jeong, Kyungjong Lee, Junghee Lee, Yeong Jeong Jeon, Jong Ho Cho, Hong Kwan Kim, Woong-Yang Park, Sang-Won Um

**Affiliations:** 1 Division of Pulmonary and Critical Care Medicine, Department of Medicine, Samsung Medical Center, Sungkyunkwan University School of Medicine, Seoul 06351, Republic of Korea; boguen86.kim@samsung.com (B.-G.K.); mn.hwang@sbri.co.kr (M.H.); gyeong.mi@sbri.co.kr (K.-M.P.); junsu.choe@samsung.com (J.C.); fresh.shin@samsung.com (S.H.S.); bh82.jeong@samsung.com (B.-H.J.); kj2011.lee@samsung.com (K.L.); 2 Division of Pulmonary Medicine, Department of Internal Medicine, Kangbuk Samsung Hospital, Sungkyunkwan University School of Medicine, Seoul 03181, Republic of Korea; 3 Samsung Genome Institute, Samsung Medical Center, Seoul 06351, Republic of Korea; kyunghee.park@samsung.com (K.P.); woongyang.park@samsung.com (W.-Y.P.); 4 Department of Obstetrics and Gynecology, Seoul St. Mary’s Hospital, College of Medicine, The Catholic University of Korea, 222, Banpo-daero, Seocho-gu, Seoul 06591, Republic of Korea; hyewon153.lee@catholic.ac.kr; 5 Department of Thoracic and Cardiovascular Surgery, Samsung Medical Center, Sungkyunkwan University School of Medicine, Seoul 06351, Republic of Korea; jhts.lee@samsung.com (J.L.); ts.yj.jeon@samsung.com (Y.J.J.); jongho9595.cho@samsung.com (J.H.C.); hkts@skku.edu (H.K.K.); 6 Department of Health Sciences and Technology, SAIHST, Sungkyunkwan University, Seoul 06351, Republic of Korea

## Error in Figures 3 and 4

In the original publication [1], there was a mistake in Figures 3 and 4 as published. The contents of Figures 3 and 4 have been swapped. The corrected Figure 3 and Figure 4 appear below. The authors state that the scientific conclusions are unaffected. This correction was approved by the Academic Editor. The original publication has also been updated.

**Figure 3:**
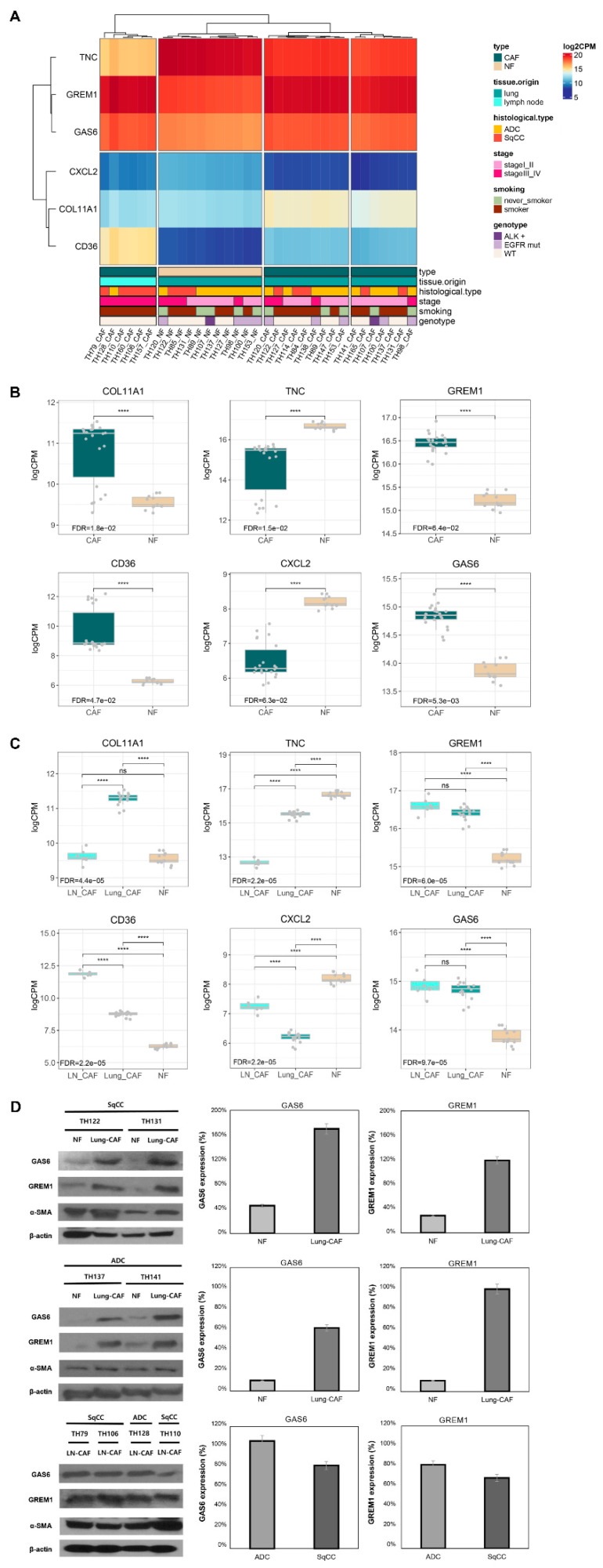
Six selected DEGs between CAFs and NFs. (**A**) Heatmap of six selected DEGs between CAFs and NFs. (**B**) Boxplot of DEGs comparing CAFs and NFs. Adjusted *p*-values (FDR) from generalized linear model. COL11A1 and TNC, known fibroblast markers; GREM1, BMP pathway; CD36, antigen processing machinery pathway; CXCL2, chemokine pathway; and GAS6, hypoxia pathway. (**C**) Boxplot of DEGs comparing LN-CAFs, Lung-CAFs, and NFs. (**D**) Western blot analysis (**left**) and densitometry-based quantification (**right**) of GREM-1 and GAS6 in LN-CAFs, Lung-CAFs, and NFs derived from squamous cell carcinomas, adenocarcinomas, and non-tumorous lungs. Adjusted *p*-values (FDR): ns, *p* > 0.05; ****, and *p* ≤ 0.0001. CAF, cancer-associated fibroblast; NF, normal fibroblast; SqCC, squamous cell carcinoma; and ADC, adenocarcinoma.

**Figure 4:**
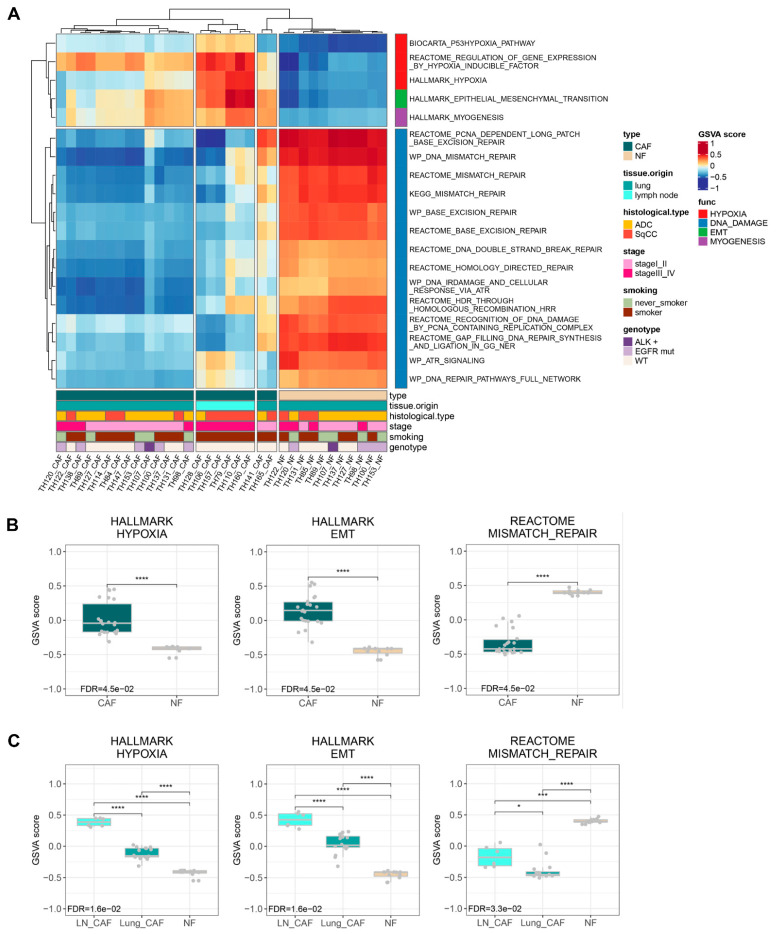
DEG sets between CAFs and NFs. (**A**) Heatmap of GSVA scores in selected DEG sets among well-known pathway databases (e.g., HALLMARK, BIOCARTA, KEGG, REACTOME, and WIKI pathway). (**B**) Boxplot of GSVA scores in selected DEG sets comparing CAFs and NFs. Gene sets related to hypoxia responses (Hallmark Hypoxia) and EMT (Hallmark EMT) were upregulated in CAFs compared with NFs (all FDR, *p* ≤ 0.0001). Conversely, the gene set related to mismatch repair (Reactome Mismatch Repair) exhibited significantly higher GSVA scores in NFs than in CAFs (FDR, *p* ≤ 0.0001). (**C**) Boxplot of GSVA scores in selected DEG sets comparing LN-CAFs, Lung-CAFs, and NFs. LN-CAFs showed higher GSVA scores for gene sets such as Hallmark Hypoxia and Hallmark EMT, compared with Lung-CAFs (FDR, *p* ≤ 0.0001). Adjusted *p*-values (FDR): *, *p* ≤ 0.05; ***, *p* ≤ 0.001; ****, and *p* ≤ 0.0001. CAF, cancer-associated fibroblast; EMT, epithelial–mesenchymal transition; NF, normal fibroblast; and GSVA, gene set variation analysis.
