# Supplementary material for: Identification of GREM-1 and GAS6 as Specific Biomarkers for Cancer-Associated Fibroblasts Derived from Patients with Non-Small-Cell Lung Cancer
Source: Cancers (Basel). 2025 Aug 30;17(17):2858. doi: 10.3390/cancers17172858 (PMC12427240; doi:10.3390/cancers17172858)
Supplement: Supplementary file 1 [file cancers-17-02858-s001.zip › Data S4.pdf]

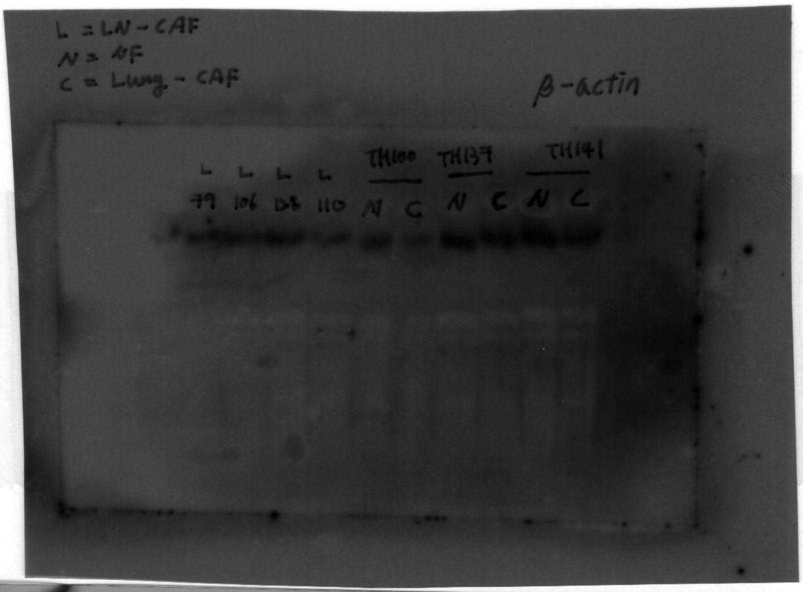
L = LN - CAF
N = NF
C = Lung - CAF
β-actin
TH100 TH137 TH141
L L L L
79 106 128 110 N C N C N C

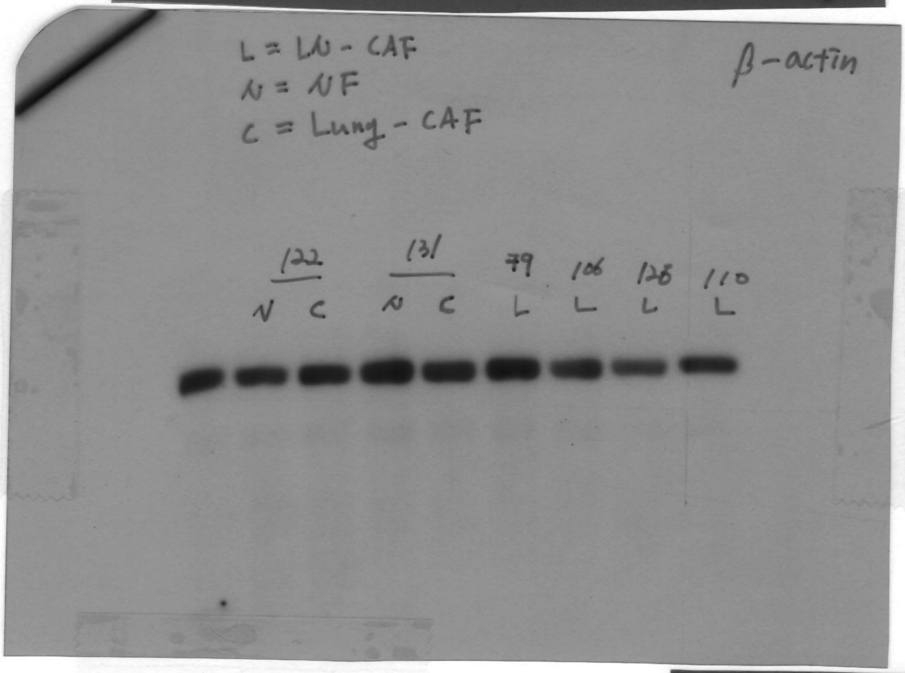
L = LN - CAF
N = NF
C = Lung - CAF
β-actin
122 131 79 106 128 110
N C N C L L L L

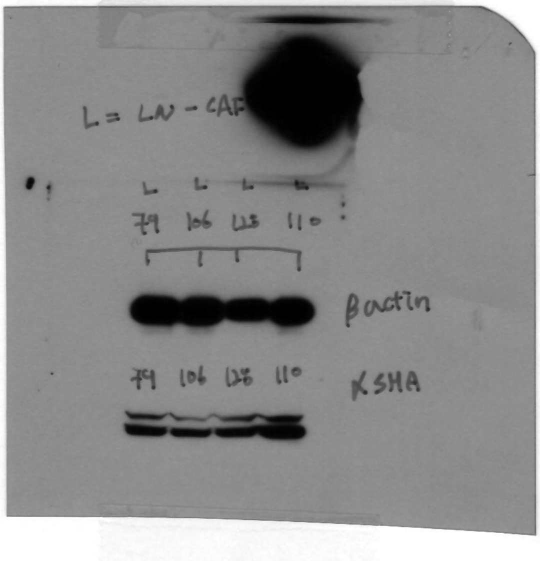
L = LN - CAF
L L L L
79 106 128 110
βactin
79 106 128 110
αSMA

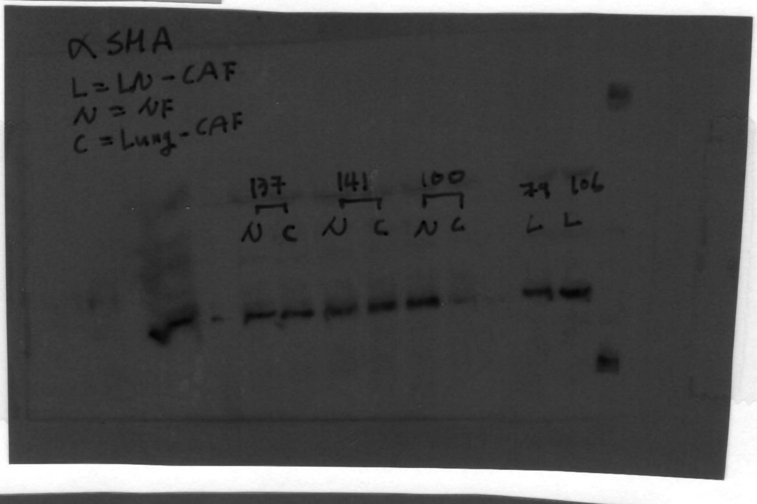
αSMA
L = LN - CAF
N = NF
C = Lung - CAF
137 141 100 79 106
N C N C N C L L

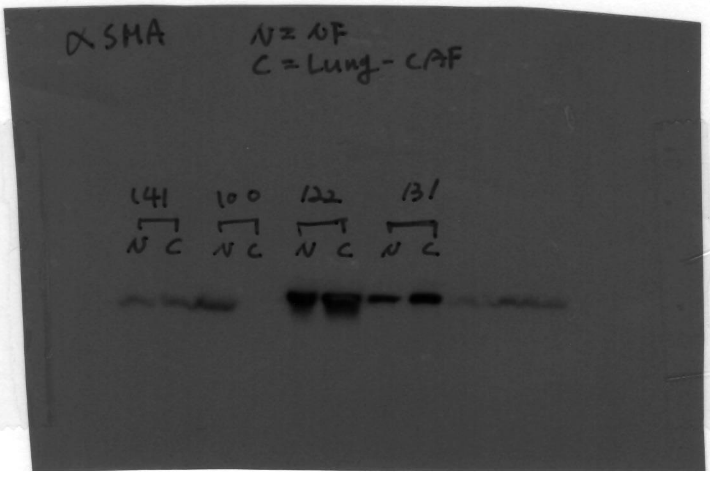
αSMA
N = NF
C = Lung - CAF
141 100 122 131
N C N C N C N C

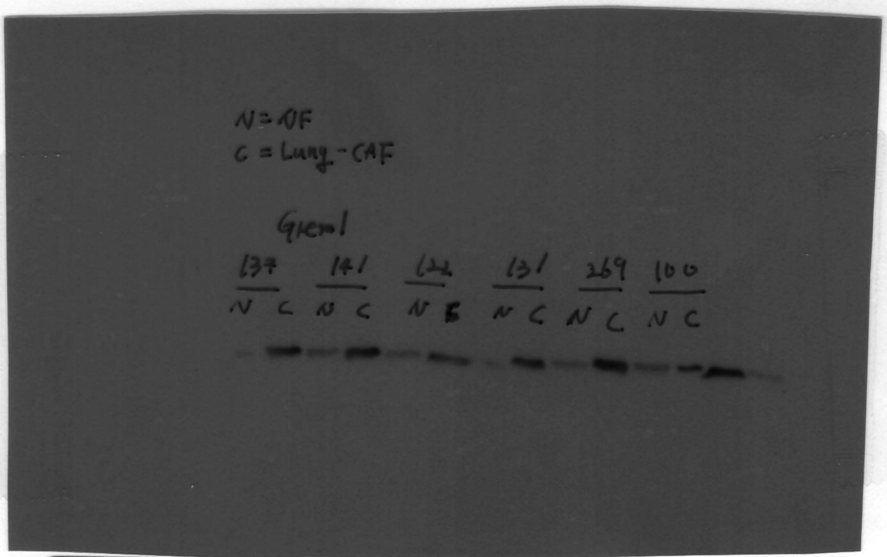
N = NF
C = Lung-CAF
Greml
137 141 122 131 269 100
N C N C N C N C N C N C

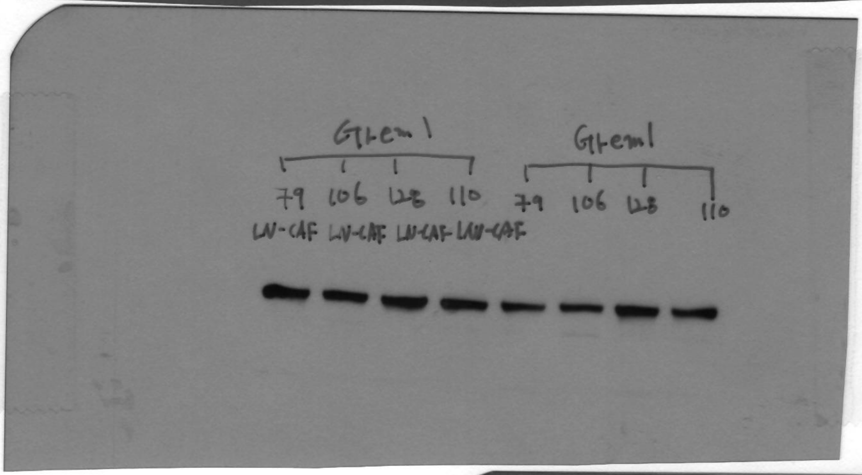
Greml
Greml
79 106 128 110 79 106 128 110
LN-CAF LN-CAF LN-CAF LN-CAF

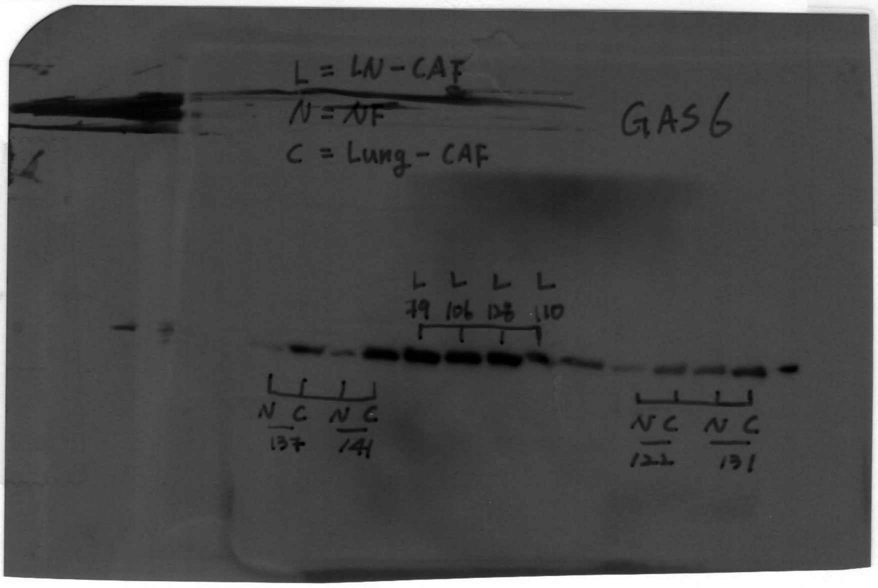
L = LN-CAF
N = NF
C = Lung-CAF
GAS6
L L L L
79 106 128 110
N C N C
137 141
N C N C
122 131

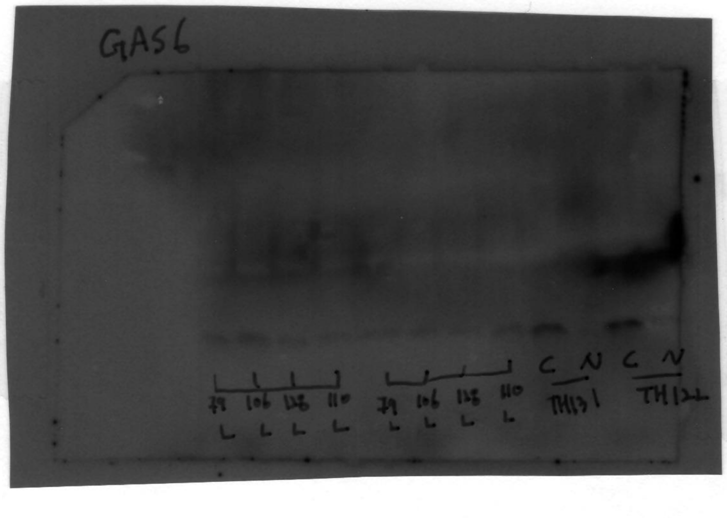
GAS6
C N C N
79 106 128 110 79 106 128 110
TH131 TH122
L L L L L L L L
