# Supplementary material for: Identification of GREM-1 and GAS6 as Specific Biomarkers for Cancer-Associated Fibroblasts Derived from Patients with Non-Small-Cell Lung Cancer
Source: Cancers (Basel). 2025 Aug 30;17(17):2858. doi: 10.3390/cancers17172858 (PMC12427240; doi:10.3390/cancers17172858)
Supplement: Supplementary file 1 [file cancers-17-02858-s001.zip › Supplemental material.pdf]

Supplemental information

- Data S1. Excel file containing the baseline characteristics of study population

- Data S2. Excel file containing the DEGs between CAFs and NFs.

- Data S3. Excel file containing the GSVA scores in selected gene sets, comparing CAFs with NFs.

-Data S4: Original version of whole Western blot for Figure 2D

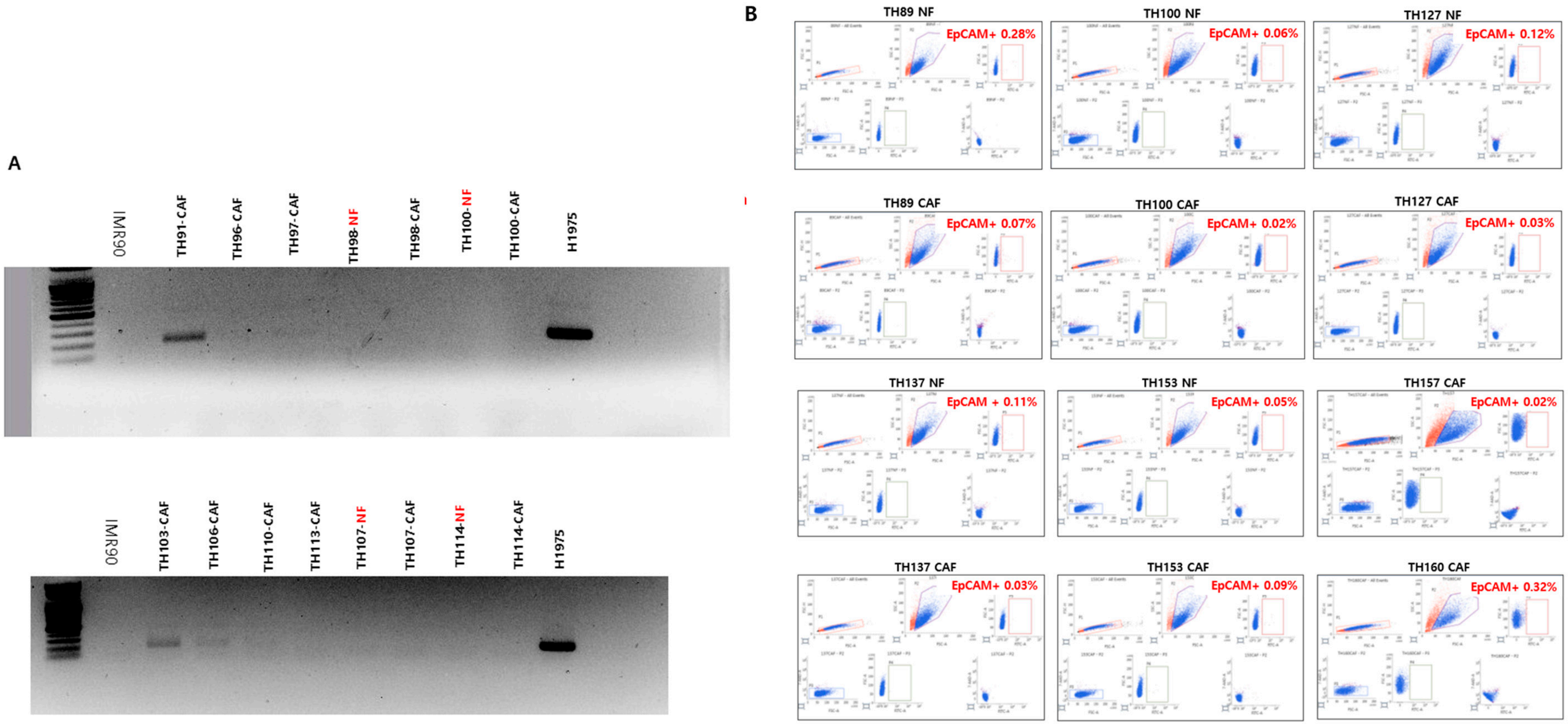

Figure S1. Representative results of RT-PCR (A) and flow cytometry (B) for the evaluation of the purity of CAFs and NFs

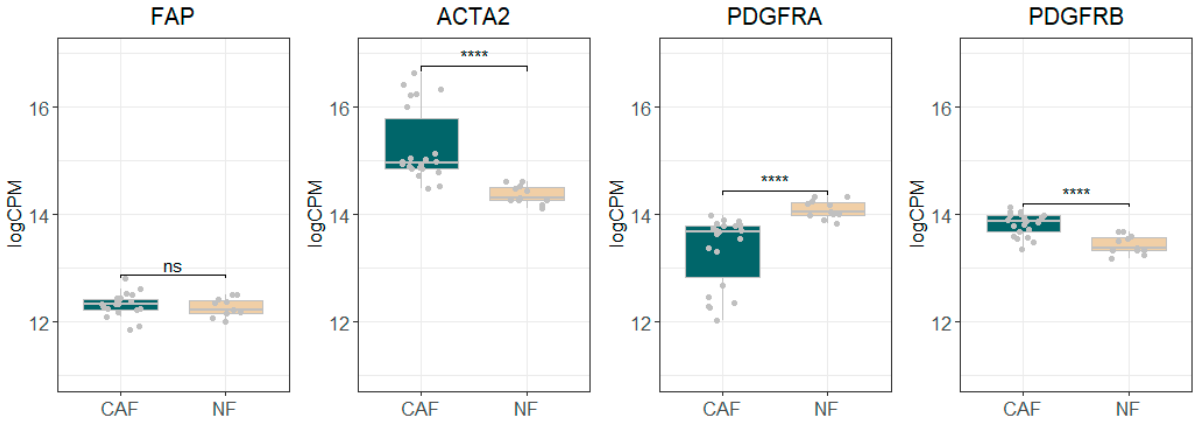

Figure S2. The expression of canonical positive fibroblast markers.

Table S1. List of primer sequences used for real-time qPCR

Name	Primer probe	Sequence	Tm (°C)
GAPDH	Forward	TGCACCACCAACTGCTTAGC	60.2
	Reverse	GGCATGGACTGTGGTCATGAG	60
CD68	Forward	CTTCTCTCATTCCCCTATGGACA	58
	Reverse	GAAGGACACATTGTACTCCACC	57.7
CD80	Forward	CTCACTATGCTGCTTCACAA	54.8
	Reverse	CAATACGGGAAACACTGCTA	54.5
IL-6	Forward	ACTCACCTCTTCAGAACGAATTG	57.7
	Reverse	CCATCTTTGGAAGGTTCAGGTTG	58.8
CD206	Forward	GCAAAGTGGATTACGTGTCTTG	57.2
	Reverse	CTGTTATGTCGCTGGCAAATG	57.3
IL-10	Forward	TACGGCGCTGTCATCGATTT	59.2
	Reverse	TAGAGTCGCCACCCTGATGT	59.3
CXCL12	Forward	GAGCTACAGATGCCCATGC	57.4
	Reverse	CTTTAGCTTCGGGTCAATGC	56.2
E-cadherin	Forward	CTGAGCTCCCTGACAAAAAT	55.63
	Reverse	GGAGGATTATCGTTGGTGTC	55.64
GAPDH	Forward	CATCATCCCTGCCTCTACTG	57.06
	Reverse	TTGGCAGGTTTTTCTAGACG	55.98

Table S2. List of antibodies used in this study.

Markers	Fluorochrome	Clone	Company	Catalogue number
Isotype control IgG1	PE		Biolegend	400111
7AAD	PE-Cy 5		Miltenyi Biotec	130-111-568
EpCAM	FITC	9C4	Biolegend	324204
